# The Effect of Antioxidant and Anti-Inflammatory Capacity of Diet on Psoriasis and Psoriatic Arthritis Phenotype: Nutrition as Therapeutic Tool?

**DOI:** 10.3390/antiox10020157

**Published:** 2021-01-22

**Authors:** Pelagia Katsimbri, Emmanouil Korakas, Aikaterini Kountouri, Ignatios Ikonomidis, Elias Tsougos, Dionysios Vlachos, Evangelia Papadavid, Athanasios Raptis, Vaia Lambadiari

**Affiliations:** 1 Rheumatology and Clinical Immunology Unit, Fourth Department of Internal Medicine, Attikon Hospital, Medical School, National and Kapodistrian University of Athens, 12462 Athens, Greece; pelkats@gmail.com; 2 Second Department of Internal Medicine, Attikon University Hospital, Medical School, National and Kapodistrian University of Athens, 12462 Athens, Greece; mankor-th@hotmail.com (E.K.); KaterinaK90@hotmail.com (A.K.); atraptis@med.uoa.gr (A.R.); 3 Second Cardiology Department, Attikon University Hospital, Medical School, National and Kapodistrian University of Athens, 12462 Athens, Greece; igikon@med.uoa.gr; 4 Department of Cardiology, Heart Failure and Preventive Cardiology Section, Ygeia Hospital, 12462 Athens, Greece; ITsougkos@hygeia.gr; 5 Independent Researcher, General Physician, 16451 Athens, Greece; vlachos.dion@gmail.com; 6 Second Department of Dermatology and Venereology, University of Athens Medical School, 12462 Athens, Greece; epapad@med.uoa.gr

**Keywords:** inflammation, immunity, nutrients, antioxidants, diet, obesity, psoriasis, arthritis, fatty acids

## Abstract

Chronic inflammation and increased oxidative stress are contributing factors to many non-communicable diseases. A growing body of evidence indicates that dietary nutrients can activate the immune system and may lead to the overproduction of pro-inflammatory cytokines. Fatty acids as macronutrients are key players for immunomodulation, with n-3 polyunsaturated fatty acids having the most beneficial effect, while polyphenols and carotenoids seem to be the most promising antioxidants. Psoriasis is a chronic, immune-mediated inflammatory disease with multifactorial etiology. Obesity is a major risk factor for psoriasis, which leads to worse clinical outcomes. Weight loss interventions and, generally, dietary regimens such as gluten-free and Mediterranean diet or supplement use may potentially improve psoriasis’ natural course and response to therapy. However, data about more sophisticated nutritional patterns, such as ketogenic, very low-carb or specific macro- and micro-nutrient substitution, are scarce. This review aims to present the effect of strictly structured dietary nutrients, that are known to affect glucose/lipid metabolism and insulin responses, on chronic inflammation and immunity, and to discuss the utility of nutritional regimens as possible therapeutic tools for psoriasis and psoriatic arthritis.

## 1. Introduction

Chronic inflammation refers to a series of pathophysiological dysregulations which eventually result in a sustained, increased production of pro-inflammatory cytokines and oxidative stress. In recent decades, numerous studies have focused on the association between the inflammatory process and the development of chronic, non-communicable diseases (NCD), such as obesity, diabetes mellitus, cardiovascular diseases, cancer and autoinflammatory diseases such as rheumatoid arthritis and psoriasis [1]. Apart from genetic and environmental factors, nutrition has emerged as a potential modulator of immunological and inflammatory responses [2]. Dietary patterns, such as the Mediterranean diet, which encompass a high intake of fruits and vegetables and a low proportion of fat and sugars, have been proposed to ameliorate chronic inflammation and reduce the incidence of NCD [3]. However, as every diet comprises many different nutrients from different food sources, extensive research has been conducted on the specific nutrients that constitute an everyday dietary regimen, which include macronutrients, namely proteins, fats and carbohydrates, and micronutrients, such as vitamins, trace elements and antioxidants such as polyphenols and carotenoids, which, in turn, can have a different impact depending both on their daily intake and food origin [4].

Psoriasis is one of the most prevalent autoinflammatory diseases worldwide, with an incidence of 2–3% in Europe and North America [5]. Its etiology is considered multifactorial, and it is characterized by the dysregulation of the innate and adaptive immune systems, with the activation of T helper (Th)-1 and Th-17 T cells leading to an increased production of inflammatory cytokines such as interleukins (IL) IL-1, IL-6, IL-23, IL-22, IL-17, and IL-33, tumor necrosis factor alpha (TNF-α), and interferon-gamma (IFN-γ) [6,7]. In this cascade, inflammation plays a cardinal role by promoting hyper-proliferation and angiogenesis, leading to the typical skin lesions and the articular involvement of psoriatic arthritis [8]. Since the report by Späh [9], where the idea of a potential common inflammatory pathway between psoriasis and atherosclerosis was first introduced, the link between psoriasis and chronic inflammation has been highlighted in many studies [10,11,12]. As inflammation is modulated by nutrition, it comes as no surprise that the impact of diet on the incidence and severity of the disease as well as on treatment response has been a matter of extensive research [13]. In this review, the role of macro- and micronutrients in inflammation and immunity will be described. Then, we discuss the most recent research data on the impact of obesity on psoriatic disease, and whether and how different dietary patterns and food components may prove beneficial in the course of the disease and emerge as possible therapeutic options along with the conventional pharmacologic treatment.

## 2. Dietary Nutrients in Inflammation and Immunity

### 2.1. Macronutrients

#### 2.1.1. Proteins

The effects of protein intake on metabolic derangement and inflammation are rather conflictive, and large-scale studies on the association between long-term habitual protein intake and circulating inflammatory biomarkers are lacking. In the Diet, Obesity, and Genes (DiOGenes) study [14], patients on low-protein diets achieved a −0.25 mg/L greater reduction in high-sensitivity C-reactive protein (CRP) (used as a disease severity and activity index) than those in high-protein diets after 8 weeks. In another study where participants were on an energy-restricted diet, those on the high-protein group demonstrated higher values of CRP, IL-1, TNF-α and plasminogen activator inhibitor-1 (PAI-1), however, this observation was valid only for meat protein and not fish or plant protein [15]. In a similar manner, in a 6-week ad libitum high animal compared with high plant protein (30% energy) diet in overweight or obese individuals with nonalcoholic fatty liver disease (NAFLD) and type 2 diabetes mellitus (T2DM), no differences in IL-6 or monocyte chemoattractant protein 1 (MCP-1) was observed, while decreases in IL-18 and TNF-α were observed only in the high animal and in the plant protein group, respectively, suggesting that not only the amount, but also the source of protein intake is important in the inflammation process [16]. In the same notion, a study in 482 women demonstrated a positive co-relation of the amount of red meat consumption and CRP levels [17], and a cross-sectional study with 553 adults showed that the consumption of processed meat showed positive association with inflammation markers such as IL-6 and TNF-α [18]. On the other hand, dietary patterns rich in plant protein sources or dairy products have generally showed a favorable impact on the metabolic environment [19,20].

#### 2.1.2. Lipids

Dietary lipids include cholesterol and fatty acids (FAs), with the latter playing a crucial role in inflammation and immunity. FAs can be divided in two separate groups [21]. The first one consists of saturated fatty acids (SFAs), such as palmitic acid and lauric acid, while the second one comprises monounsaturated (MUFAs), such as n-9 oleic acid (OA), and polyunsaturated (PUFAs) fatty acids, such as a-linolenic acid (ALA) and linoleic acid (LA), depending on the number of double bonds they contain. SFAs are primarily found in meat, dairy products, palm and coconut oil and processed foods which are abundant in “Western-type” diets and have been associated with increased inflammation in a number of studies [22,23,24,25,26]. In a retrospective cohort with elderly patients [23], lower ingestion of SFAs was associated with the reduction of CRP in lean and overweight subjects, and in a three-week randomized crossover study on 15 overweight women, the replacement of an SFA-rich diet (42% of the total caloric value-TCV) by a PUFA-rich diet (40% of the TCV) resulted in a decrease in CRP and IL-8 levels [24]. In a study by Lyte et al. [25], where healthy adults consumed one of four types of isoenergetic meals, postprandial serum endotoxin concentration was increased after the SFA meal; similarly, in a study where healthy, lean and obese adults consumed high-PA (HPA) and low-PA/high-oleic-acid (HOA) diets for 3 weeks, lower secretion of interleukin (IL)-1β, IL-18, and TNF-α by peripheral blood mononuclear cells (PBMCs) was observed in the HOA group [26]. SFAs activate dendritic cells, which results in the endocytosis of Toll-like receptor (TLR)-4 and the secretion of pro-inflammatory cytokines such as IL-1β and reactive oxygen species (ROS) [27]. Activation of TLR-2 results in reduced suppressive capacity of T-regulatory (Treg) cells, which are also converted into a Th17-like phenotype, leading to a dysregulated pro-inflammatory T-cell response [28]. SFAs are also related to inflammasome-mediated inflammation, as it was shown in a mouse model, where animals fed with a high-fat diet (45% PA) showed enhanced TLR4-dependent NLRP3-inflammasome activation and IL-1β secretion from dendritic cells [29].

PUFAs are comprised of two different categories, omega-3 (n-3) and omega-6 (n-6) fatty acids. Omega-3 FAs include α-linolenic acid (ALA) which is mainly found in plants, and eicosapentaenoic acid (EPA) and docosahexaenoic acid (DHA) found mainly in fish and seafood sources. Omega-6 FAs include linoleic acid (LA), mainly found in meat, and arachidonic acid (ARA), found in poultry and eggs [21]. Omega-3 fatty acids are generally considered to have anti-inflammatory capacities compared to omega-6 ones, although this is not always the case. As they compete for the same enzymes, it is the n-6/n-3 ratio which seems to be more significant for the maintenance of a healthy metabolic milieu, with the ideal ratio reaching up to 4:1, contrary to the usual 10:1 ratio which applies for Western-type diets [30]. In a randomized controlled trial (RCT) in healthy adults, a 4-month supplementation of n-3 fatty acids of 2.5 g/d and 1.25 g/d versus placebo resulted in a significant decrease in IL-6 levels by 10% and 12% in the low and high dose n-3 FAs groups, respectively, compared to a 36% increase in the placebo group [31]. Similarly, in a 12-week study, subjects who received n-3 supplementation showed a 14% decrease in lipopolysaccharide (LPS) stimulated interleukin 6 (IL-6) production, while a decrease in the n-6:n-3 ratio led to reductions in stimulated IL-6 and TNF-α production [32]. In a study by Dangardt et al. [33], the supplementation of flaxseed flour, a source of omega-3 fatty acids, to morbidly obese subjects, led to decreased CRP and serum amyloid A (SAA) concentrations. The consumption of EPA (180 mg/d) and DHA (120 mg/d) for six weeks was associated with a reduction in CRP in overweight patients [34], and a similar anti-inflammatory pattern was observed in a study with patients with T2DM, where the supplementation of high doses of n-3 PUFAs led to lower concentrations of IL-2 and TNF-α [35]. However, some other studies have failed to support any beneficial effect of n-3 supplementation on inflammatory biomarkers, with this discrepancy being attributed to the relatively low supplemented daily dose, which did not exceed 6.6 g/d [36,37,38]. 

Omega-3 FAs’ anti-inflammatory effects are mainly attributed to the interaction with the G-protein coupled receptor 120 (GPR120), expressed on macrophages, which leads to decreased TLR4-dependent and LPS-mediated cyclooxygenase-2 (COX-2) activation and subsequent prostaglandin release [39], while another action on macrophages includes the inhibition of the NOD-like receptor protein 3 (NLRP3) inflammasome activation [40]. By inhibiting nuclear factor kappa-B (NF-κB) activity, they decrease the production of pro-inflammatory cytokines such as intracellular adhesion molecule (ICAM)-1, vascular cell adhesion molecule (VCAM)-1 and E-selectin [41]. Apart from their actions on innate immunity, n-3 PUFAs are crucial in the function of adaptive immunity as well, acting on T cells by regulating the janus kinases—signal transducer and activator of transcription proteins (JAK-STAT) pathway by decreasing JAK1 and JAK3 phosphorylation and, thus, leading to inhibition of STAT5 phosphorylation [42]. Finally, n-3 PUFAs lead to a reduced production of TNF-α, IL-2, IL-4 and IL-10, often in a dose-dependent manner [43].

#### 2.1.3. Carbohydrates and Fiber

The amount, quality and dietary source of carbohydrates are the factors which determine their inflammatory capacity. In a study by Liu et al. on 244 healthy women, a strong positive association between a high dietary glycemic load (GL) and high-sensitive CRP (hsCRP) was demonstrated [44], and similar were the results in a study on 511 elderly subjects, where, after 1 year of follow-up, a high-glycemic index (GI) or high-GL diet led to an increase in IL-6 and TNF-α levels, along with a concomitant decrease in leptin and adiponectin levels [45]. A hyperglycemic diet (59–67% of TCV) led to higher CRP concentrations [46] and, in turn, a hypoglycemic diet (10–13% of TCV) led to decreased IL-6 levels [47], although such results were not confirmed in some other reports such as that of O’Brien et al., where decreases in CRP and SAA levels were attributed to weight loss and not the carbohydrate content of the diet per se [48]. The role of carbohydrate quality was highlighted in a study by Neuhouser et al. [49], where the low-GL diet reduced CRP compared to the high-GL diet in 80 participants, despite the fact that the diets were isocaloric. An excellent example of the importance of the carbohydrate source are fibers, which are mostly complex carbohydrates. A diet with a fiber content of 30 g/d led to a significant reduction in hsCRP [50]. A similar anti-inflammatory effect was shown in a study where 105 individuals were assigned to one of the three energy-restricted diet groups receiving rice bran, rice husk powder and control (low-calorie only) diet for 12 weeks, with the intervention groups demonstrating decreases in hsCRP and IL-6 compared to controls [51]. In general, fibers slow down carbohydrate uptake and hamper the absorption of dietary lipids [52]. In addition, certain nondigestible fibers are fermented to short-chain FAs (SCFAs), namely acetate, propionate, and butyrate, which exert anti-inflammatory effects by activating GPRs, inhibiting the release of molecules such as TNF-α, IL-1 and nitric oxide (NO), repressing NF-κΒ expression and promoting Treg cell differentiation and diversity of the gut microbiota [52,53,54,55,56].

### 2.2. Micronutrients

Micronutrients are essential elements which are found in very small quantities in the human body and include vitamins, minerals and trace elements. They are scavengers of ROS and they can limit the generation of free radicals and inflammatory molecules, serving as potent antioxidants [4]. Below, research data about the role of the main micronutrients in inflammation is summarized.

#### 2.2.1. Polyphenols

Polyphenols are the most abundant antioxidants in diet, with their main sources being fruits, vegetables, red wine, nuts, green tea, and olive oil. Many studies have pointed out the beneficial effect of a diet rich in polyphenol sources on inflammation and human health [57]. In 24 obese adults, the consumption of 46 g of grape powder for 3 weeks led to a significant decrease in low-density lipoprotein cholesterol (LDL-C) and cholesterol levels and enhanced immune function [58], and a similar result was reproduced in a study by Ravn-Haren et al. with the supplementation of apple or apple pomace [59]. Consumption of cooked purple potato (200 g/day containing 288 mg anthocyanins) for 14 days reduced pulse wave velocity, an indicator of endothelial dysfunction, compared with white potato in healthy humans [60]. In 62 patients with at least one cardiovascular disease (CVD) factor, consumption of 330 mL/day bilberry juice resulted in a reduction in CRP, IL-6 and IL-15 levels [61]. In women with metabolic syndrome, the consumption of freeze-dried strawberries (FDS) for 8 weeks decreased LDL and vascular cell adhesion molecule 1 (VCAM-1), and similar were the results in another cohort with T2DM female patients [62]. Since fruits are also rich in other components, such as vitamins and fiber, some studies in animal models have used purified polyphenol extracts to address their role more specifically. Yang et al. supplemented polyphenol extracts of the sea buckthorn berry to rats fed a high-fat diet (HFD), reducing TNF-α, IL-6, endothelial nitric oxide synthase (eNOS), ICAM-1 and lipid levels [63]. A diet enriched with 1% blueberry reduced aortic atherosclerotic lesions area in ApoE^−/−^ mice, with a concomitant increase in the antioxidant activity of superoxide dismutase (SOD) 1, SOD2 and GRx (enzymes which catalyze the dismutation of the superoxide radical) and reduced lipid peroxidation [64].

Resveratrol, a flavonoid mainly found in red wine, decreases the concentration of TNF-α by activating SIRT-1 (promoting autophagy), eNOS (increasing NO production), and Nrf2 (inhibiting the NF-κB pathway and proinflammatory cytokine production) pathways, decreases the expression of ICAM-1 and VCAM-1 via inhibition of the NF-κB pathway activation and reduces the formation of foam cells via inhibition of NADPH oxidase-1 in mouse macrophages [65,66,67]. Quercetin, a flavonol found in sources such as red onions, capers, apples and nuts, reduces inflammation and oxidative stress in human and animal models. Mechanisms include reduction of the formation of foam cells by increasing the expression of PPAR-receptors and ATP-binding cassette transporter (ABCA1), suppressing of leukocyte recruitment and decreasing the production of Th-1 derived IFN-γ, among others [68]. Curcumin, a flavonoid that is mainly found in turmeric, curry spice, and ginger, has been shown to decrease SOD, malondialdehyde (MDA) and CRP in patients with metabolic syndrome and IL-1β, TNF-α, ICAM-1 and VCAM-1 in mice, through the inhibition of the TLR4 signaling pathway [69,70]. However, a serious disadvantage of the aforementioned studies is that the beneficial results were achieved at pharmacological doses that highly exceed normal daily food intake, a fact which renders the utility of antioxidants in everyday clinical practice questionable. 

Another food source rich in phenolic compounds is olive oil, which is a basic component of Mediterranean diet. Its anti-inflammatory capacities are attributed not only to its favorable lipid profile, with oleic acid being its major fraction (55–83%), but also to its minor compounds, with the phenols oleuropein and hydroxytyrosol standing out nutritionally [71]. The beneficial effect of olive oil and, especially, extra virgin olive oil (EVOO) has been demonstrated in a number of studies [72,73,74,75,76,77]. In blood platelets taken from 5 healthy subjects, the supplementation with 0.2 mg/mL EVOO with or without catalase reduced the activation of Nox2 and H_2_O_2_ production [78]. In 33 hypercholesterolemic patients, EVOO enriched with polyphenols led to an increase in HDL and antioxidant capacity [79], and in cells of patients with ulcerative colitis, cells stimulated with 3 mM oleuropein demonstrated reduced production of TNF-α, IL-1β, IL-17 and COX-2 [80]. In individuals with impaired fasting glucose receiving a daily meal containing 10 g EVOO, postprandial circulating LPS stabilized, along with the oxidation of LDL and NADPH oxidase 2 (Nox2) [81]. In a sub-study associated with the Prevención con Dieta Mediterránea (PREDIMED) trial, individuals receiving Mediterranean diet plus EVOO showed a reduction in IL-6, VCAM-1, ICAM-1 and LDL, and an increase in HDL [82]. Similar results were reproduced in studies regarding the association of olive oil with cardiovascular events [83,84,85,86], as well as in animal models [87,88]. The phenolic compounds in EVOO are capable of diminishing LDL oxidization and possess potent antioxidant properties, with oleuropein also having anti-proliferative properties [7]. 

#### 2.2.2. Carotenoids

Carotenoids are mainly found in plants, bacteria and fungi, with the most important being carotenes and xanthophylls [89]. Carotenoids serve primarily as potent antioxidants, being able to quench radicals and singlet oxygen, interact with nuclear receptors RAR/RXR (retinoic acid receptor/retinoid X receptor) to enhance immune pathways, and inhibit the pro-inflammatory NF-κB pathway [90]. In an RCT by Yoshida et al. with nonobese humans [91], supplementation of astaxanthin (a xanthophyll found mainly in seafood) for 12 weeks improved HDL-C, TG, and adiponectin levels. In patients with peripheral artery disease, supplementation with orange and blackcurrant juice for 4 weeks led to a reduction in CRP and plasma fibrinogen [92]; similarly, MDA and 8,12-isoprostane F2a-VI levels were inversely associated with concentrations of individual carotenoids [93]. Lutein, found mainly in broccoli and spinach, has a beneficial impact on arterial stiffness and decreases IL-6, TNF-α and prostaglandin E2 (PGE2) [94]. Beta-carotene, found mainly in carrots and tomatoes, reduces the expression of IL-1β and VCAM-1 in LDL receptor knockout mice [95]. On the other hand, positive associations between lycopene and F2-isoprostane or MDA levels have been shown in large-scale human studies, which were attributed to the fact that lycopene intake came mainly from pasta sauce, ketchup and fast-food consumption and not from tomatoes, its main natural source [96,97]. In patients with cystic fibrosis, beta-carotene decreased MDA levels at the dose of 1 mg/kg/d, but not at 10 mg/d [98]. In a study with healthy subjects receiving supplementation with 5, 10, 20 or 40 mg/d beta-carotene for 5 weeks, MDA was reduced only in the highest group [99], while in another cohort, supplementation with either 15 or 120 mg/d beta-carotene led to similar reductions in lipid peroxide levels [100]. In conclusion, evidence from large-scale trials about the clinical significance of carotenoids remains contradicting.

#### 2.2.3. Vitamins

Vitamin A is present both in plants (carrots and red peppers) as carotenoids and in eggs, liver and milk as retinol, with both forms being processed to its active form, which is retinoic acid. Retinoic acid promotes phagocytosis and activation of natural killer (NK) T-cells and, therefore, vitamin A deficiency has been associated with defective immune responses [101,102]. Vitamin C is a potent ROS scavenger and stimulates neutrophil apoptosis and T-cell maturation, and its increased dietary intake has been associated with lower levels of CRP and PAI-1 [103,104,105]. Vitamin C is also indispensable for the antioxidant actions of vitamin E, which contributes to T-cell and NK cells activity. In humans, vitamin E supplementation has been shown to reduce pro-inflammatory cytokines IL-1β, IL-6 and TNF-α by stimulating the production of cyclic adenosine monophosphate (cAMP) [106]. Vitamins C and E are associated with a reduction in coronary artery disease, but their supplementation in high pharmacological doses seems to bear no additional benefit [107].

Despite being best renowned for its role in calcium homeostasis, vitamin D also has immunomodulatory effects, with calcitriol being necessary for T-cells function [108]. In vitro studies showed that vitamin D3 can lead to decreased expression of CD40, CD80, and CD86, and enhanced IL-10 secretion [109]. However, in the VITAL study, where vitamin D3 (cholecalciferol) and n-3 FAs were supplemented in 25,871 participants with a median follow-up of 5.3 years, no differences in the incidence of invasive cancer or cardiovascular events were shown compared to the placebo group [110]. Another concern about vitamin D is the fact that its plasma levels are affected by various determinants. Higher amounts of sun exposure have been associated with a lower risk of vitamin D deficiency; on the other hand, excessive sun exposure is the primary risk factor for skin cancer and generally seems to have a detrimental effect on many cutaneous diseases such as psoriasis [111,112]. Indeed, the optimal level of sun exposure to maintain an adequate vitamin D status is yet to be determined, while a number of other factors such as alcohol intake, physical activity and genetic polymorphisms play an important role; in fact, dietary intake seems to be of little significance [113,114]. Finally, regarding the B-complex vitamins, a high B6 intake was reversely associated with plasma CRP [115]. In patients with B12 deficiency, methylcobalamin administration improved the CD4/CD8 ratio and suppressed NK cell activity [116]. Chambers et al. [117] showed that supplementation with 5 mg/d of folic acid and 1 mg/d of B12 (typical daily intake: about 500 mcg/day and 3.4 mcg/day, respectively) for eight weeks significantly improved endothelial dilatation, and a similar result was also demonstrated in a 7-year RCT where this combination along with high doses of B6 was provided [118]. However, such high doses which cannot readily be implemented in everyday practice doubt the clinical significance of these results. 

#### 2.2.4. Trace Elements

Selenium exerts its antioxidant capacities through its incorporation in antioxidant enzymes called selenoproteins, including glutathione peroxidase (GPx), selenoprotein P, and thioredoxin reductase [119]. Despite, however, having a clear role in the enhancement of the immune system, studies have not proven that supplementation with selenium decreases the risk for cardiovascular mortality [120]. On the other hand, research data about zinc seem to be more consistent. Apart from its role in T-cell activation, zinc is a cofactor in a range of antioxidant enzymes, especially SOD and the anti-inflammatory SMAD proteins [121]. Low levels of zinc cause increased oxidative stress in endothelial cells in vitro [122]; in vivo, supplementation of zinc in diabetic mice reduced aortic tunica media thickness, VCAM-1 and PAI-1, and increased the expression of Nrf2 and metallothioneins in the aorta, both being powerful antioxidants [123]. Once again, however, it should be noted that the doses administered were extremely higher than the usual daily intake.

## 3. Diet and Psoriasis: Obesity, Dietary Regimens and Promising Therapeutic Targets

Psoriasis is a multifactorial disease, attributed to both genetic and environmental factors. Among the latter, many studies have suggested that nutrition can play a crucial role both in disease pathogenesis and treatment through its effects on chronic inflammation obesity has been recognized as a major risk factor for psoriasis development and progression, and weight loss regimens, together with other dietary interventions such as gluten-free diet or Mediterranean diet seem to offer substantial beneficial results in the course of the disease. Below, research data about the association of psoriasis and different dietary regimens will be discussed, along with possible suggestions and implication for treatment options. 

### 3.1. Obesity

#### 3.1.1. Obesity and Psoriasis: A Bidirectional Association

A growing number of studies have highlighted the association between obesity and psoriasis [124,125,126,127,128,129,130,131,132,133]. The prevalence and incidence of psoriasis is higher among patients with obesity, while obesity is an important predisposing factor for psoriasis onset, progression and severity. Moreover, obesity exerts a negative impact on the treatment of psoriasis and increases the adverse effect of anti-psoriatic drugs [134,135,136,137,138]. A cross-sectional study by Herron et al. pointed out that obesity had a higher prevalence among psoriatic patients compared to the non-psoriatic population and highlighted that body weight was not increased at the onset of the disease but rather during its course, implying that obesity was a consequence rather than a causal factor [124]. The Nurses’ Health study was the first large-scale, prospective study which pointed out that increased BMI (Body Mass Index) and weight gain are strongly associated with psoriasis, with a relative risk of 1.63 (95% CI, 1.58–2.61) for BMI ≥ 35.0 compared to controls [130]. A case-control study with 373 patients by Wolk et al. showed that obesity is associated with a two-fold increased risk of psoriasis compared to individuals with normal body weight. In addition, it was the first study investigating BMI as a risk factor for psoriasis severity, indicating that for each one-unit increment in BMI, the risk for psoriasis severity (assessed by Psoriasis Area Severity Index—PASI score) increased by 7% [131]. In accordance with these results, another study by Murray et al. supported that patients with psoriasis and particularly women are more likely to have increased BMI compared to same-gender full siblings, and a positive correlation was demonstrated between obesity and disease severity as determined by body surface area (BSA) and the Physician’s Global Assessment (PGA) [127]. Similarly, a case-controlled study by Naldi et al. showed that overweight and obese patients have an odds ratio (OR) of 1.6 and 1.9, respectively, for psoriasis compared to controls [128]. In the more recent HUNT study with 33.734 patients, Snekvik et al. prospectively examined the association between development of psoriasis and BMI, waist circumference, waist-to-hip ratio and 10-year weight gain. Patients with obesity and increased waist circumference have an almost two-fold risk for developing psoriasis, with relative risks being 1.87 and 1.95, respectively. Moreover, subjects with a body weight increase >10 kg had an RR of 1.72 [129]. The same factors were examined in a recent meta-analysis, including 7 prospective studies and 17.637 patients; for each 5 units increase in BMI and for each 5 kg of weight gain, the relative risk of psoriasis increased by 19% and 11%, respectively. Moreover, for each 10 cm increase in waist circumference and 0.1 unit increase in waist-to-hip ratio, the relative risk for psoriasis rose by 24% and 37%, respectively [132]. A previous meta-analysis, including 13 retrospective case-control and 3 prospective case-control studies, showed a relative risk of 1.66 (95% CI 1.46–1.89) for obesity among patients with psoriasis compared with controls and, even more importantly, a hazard ratio of 1.18 (95% CI: 1.14–1.23) for new onset obesity among psoriasis patients, indicating again the double role of obesity both as a cause and a repercussion of psoriatic disease [133].

#### 3.1.2. A Common Inflammatory Pathway 

Obesity and psoriasis represent chronic inflammatory states, and many recent studies have focused on the complicated role of visceral fat, which acts as an endocrine organ and releases a number of pro-inflammatory cytokines and adipocytokines resulting in immune dysregulation and low-grade inflammation [139]. Central obesity is associated with increased visceral fat, where activated macrophages stimulate adipocytes to produce pro-inflammatory molecules such as TNF-α, IL-1, IL-6 and IL-8 [140]. Adipocytokines, which are secreted not only by adipocytes but also by macrophages in adipose tissue, contribute equally to the inflammation process [141]. Leptin is an adipocytokine which ameliorates energy expenditure by inducing lipolysis and inhibiting liver lipogenesis [142]. It is well known that obesity is characterized from hyperleptinemia and leptin resistance [142,143,144]. Interestingly, increased leptin levels have also been detected in patients with psoriasis independently of BMI. Apart from aggravating the inflammatory cascade, leptin also induces keratinocyte proliferation, which is a crucial step in the development of the characteristic skin lesions in psoriasis [142,143]; at the same time, it drives T cells toward the Th-1 phenotype. Adiponectin, another adipocytokine which is produced by white adipose tissue (WAT), exerts an anti-inflammatory action by blocking the secretion of TNF-α, IL-6, IL-17 and IL-1, enhancing the secretion of IL-10 and downregulating VCAM-1 and ICAM-1 [145,146]. It should be noted, however, that the evidence regarding the role of adiponectin in psoriasis is inconsistent. A recent meta-analysis of 63 studies, including 2876 psoriasis patients and 2237 healthy controls, showed that patients with psoriasis have decreased levels of adiponectin; on the contrary, another meta-analysis with 521 cases and 482 controls indicated no statistically significant difference in the levels of adiponectin and high-molecular weight adiponectin levels between the two groups [141,147]. 

Omentin-1 is another adiponectin with anti-inflammatory properties by inhibiting TNF-α. Levels of omentin-1 are decreased in obesity, however, an inverse association with the incidence of psoriasis has not been firmly established [141]. Even more interestingly, consistent results have been shown for resistin, which seems to play a key role in the pathogenesis of the disease through an increase in secretion of pro-inflammatory cytokines such as IL-6, IL-12 and TNF-α via activation of nuclear factor-B signal pathway and is also increased in obesity and other chronic inflammatory states [148,149,150]. In a recent meta-analysis of nine case-control studies, containing 421 psoriasis patients and 348 healthy controls, serum resistin levels were higher in psoriasis patients of both Asian and Caucasian origin [151]. Even more interestingly, a study by Kyriakou et al. indicated diminished blood concentrations of resistin after treatment for psoriasis compared to their initial levels [152]. 

#### 3.1.3. Obesity and Anti-Psoriatic Treatment

Research data have shown that a high BMI diminishes the effectiveness of pharmacological treatment [134,138]. Obesity can also modify pharmacokinetics of anti-TNF and other biologic agents, leading to increased drug clearance, shorter half-life and lower serum trough drug concentrations [132]. A cohort study from Italy including patients who received systemic treatment for plaque psoriasis for the first time showed that the percentage of patients achieving reduction of PASI score >75% was 30% lower in obese patients compared to individuals with normal BMI [134]. According to an observational cohort study based on Danish and Icelandic registries, BMI > 30 is associated with higher psoriasis activity at baseline and reduced drug response and treatment adherence (HR:1.85) [135]. In a study by Bardazzi et al., among 33 patients receiving biological agents, patients who put on weight during the 8-month follow-up did not achieve PASI 50, while patients who had a stable weight presented variable response to treatment and those who decreased their weight achieved PASI 90 or PASI 75, even when not responding initially [136]. In a retrospective observational study including 110 patients on anti-TNF-α agents, Di Lernia et al. reported that after two years, the proportion of patients receiving the same treatment was only 42.21%, proposing high BMI as an independent predictor of drug failure and withdrawal [138]. Such results by single studies have been confirmed in a large meta-analysis including 54 studies and 19.372 patients, which showed that obesity is associated with 60% higher odds of inadequate response to anti-TNF treatment as compared to normal BMI; for each unit increment of BMI, there is an augmentation in odds of failure by 6.5% [137]. 

#### 3.1.4. Effect of Weight Loss Interventions in Psoriasis—A Role for Low-Calorie Diet

During recent years, it has been shown that weight loss through diet and physical exercise reduces oxidative stressors, exerts a positive effect on psoriasis severity and ameliorates the response to pharmacological treatment for psoriasis. In this notion, a significant number of studies have been conducted to investigate the effect of low-calorie diet on psoriasis severity and progression. In a randomized controlled trial with 60 patients, Jensen et al. reported that a 16-week low-energy diet (800–1000 Kcal/day) resulted in significant weight loss (mean change −15.8 Kg compared to −0.4 Kg in control groups), improvements in PASI score (mean change −2.3 vs. −0.3 in control group) and statistically significant amelioration in Dermatology Life Quality Index (a ten-question questionnaire measuring the impact of skin disease on the quality of life) in patients with BMI > 27 compared to control group [153]. These results are in accordance with another RCT by Naldi et al., where a 20-week hypocaloric diet in combination with exercise resulted in a median PASI reduction of 48.0% (95% CI, 33.3%–58.3%) in the intervention group vs. 25.5% (95% confidence intervals-CI, 18.2%–33.3%) in controls. Interestingly, the improvement of psoriasis severity in intervention group was achieved with only a slight weight loss [154]. A recent meta-analysis of six RCTs confirmed that weight loss following lifestyle interventions improves psoriasis compared with controls, with a mean change in PASI score of 2.59. Apart from weight loss per se, such results can be explained also by the effect of physical activity, which upregulates anti-inflammatory molecules such as IL-10 and downregulates TLR expression on monocytes, leading to more favorable immune responses. In addition, bariatric surgery, particularly gastric bypass, reduces the risk of developing psoriasis (HR: 0.52) [155].

Del Giglio et al. conducted a randomized controlled trial to investigate the effect of low-calorie diet on maintenance of psoriasis remission after 12 weeks of methotrexate treatment. After 14-weeks of low-calorie diet, the patients in intervention group did not manage to maintain a statistically significant remission rate, possibly because weight regain was progressively observed in the intervention group after week 24; however, there was a trend towards a slower rebound of psoriasis compared to control group [156]. Similarly, Mutairi et al. conducted a randomized controlled study to assess the impact of weight loss on the efficacy of biologic agents to obese patients with psoriasis. A diet intervention for 24 weeks resulted in a mean weight reduction of 12.9 ± 1.2 kg in the intervention and 1.5 ± 0.5 kg in the control group, which led to a 84% and 69% improvement in PASI score, respectively [157]. Another prospective study showed that a weight loss >5% is significantly associated with the achievement of minimal disease activity as a response to anti-TNF treatment in obese patients with psoriatic arthritis [158]. A 24-week randomized controlled trial by Gisondi et al. showed that low-calorie diet improves response to cyclosporin treatment in obese patients with moderate-to-severe psoriasis [159]. A possible explanation for the beneficial effect of weight loss on psoriasis progression is the amelioration of obesity-induced inflammation by decreasing the size and the inflammatory activity of hypertrophic adipocytes, the infiltration of adipose tissue by macrophages and the secretion of pro-inflammatory cytokines. Lifestyle intervention and weight loss have been associated with the reduction of TNF-α, IL-8, IL-6, CRP and MCP-1 levels [160]. It becomes evident, therefore, that patients with psoriasis should be encouraged to attempt healthy lifestyle changes in order to maintain normal weight. 

### 3.2. Dietary Patterns and Psoriasis

Apart from low-calorie diets, a number of nutrition strategies and dietary patterns such as gluten-free diet, Mediterranean diet and very-low-carb ketogenic diet have been proposed for weight loss achievement in patients with psoriasis. A diet rich in antioxidants can also be considered a substantial part of a comprehensive treatment regimen along with pharmacotherapy. Despite the lack of large randomized clinical trials to confirm the effect of different diets in patients with psoriasis, an abundance of research data highlights diet as a potential therapeutic target. 

#### 3.2.1. Gluten-Free Diet

There is a high prevalence of gluten sensitivity and celiac disease (CD) among patients with psoriasis. A recent systematic review concluded that patients with psoriasis have a 2.16-fold increased risk to be diagnosed with CD [161]. According to another meta-analysis from 2014, patients with psoriasis have a 2.4-fold increased risk of seropositivity for antigliadin antibodies compared to healthy controls and, even more importantly, elevated antibody titers are associated with psoriasis severity. Furthermore, it was pointed out that psoriasis may be associated with gluten sensitivity without celiac disease, as elevated values of CD antibodies may exist even with negative duodenal biopsy results [162]. As a result, the potential role of gluten-free diet in psoriasis has been thoroughly studied. Studies by Michaëlsson et al. showed that three months of gluten-free diet improves skin biopsy anomalies (reduction in Ki67 positive cells and tissue transglutaminase-tTG expression in dermis) and psoriasis severity (reduction of psoriasis area and PASI score from 5.5 ± 4.5 to 3.6 ± 3) in patients with psoriasis and positive IgA-AGA or IgG-AGA antibodies [163,164]. The gluten-free diet proved beneficial for seropositive patients even with a normal biopsy; this finding, however, was not confirmed in the absence of antigliadin antibodies. Similarly, a more recent study by Kolchak et al. showed that one year of gluten-free diet improves PASI score in 56% of patients with very high levels of IgA-AGA and in 36% of patients with high levels of IgA-AGA [165]. A study from De Bastiani et al. showed that a 3-month gluten-free diet resulted in significant amelioration in PASI score in all patients; the improvement was sustained in 89% of patients after six months [166]. Apart from the presence of shared genes (at-risk HLA haplotypes) which has been hypothesized to play a role in the association between CD and psoriasis, the malabsorption caused by CD and the consequent vitamin D deficiency seems to be another factor predisposing to psoriasis onset and severity. Overall, available studies suggest that gluten-free diet results in clinical improvement of psoriasis in patients with positive serum markers, with or without celiac disease; however, large randomized controlled trials have not yet been conducted. Based on this evidence, the Medical Board of the National Psoriasis Foundation recommends a trial of a three-month gluten-free diet in adults with psoriasis and positive serum markers for gluten sensitivity as an add-on strategy to the standard therapies [167].

#### 3.2.2. Mediterranean Diet

The Mediterranean diet (MD) is a healthy eating pattern which is characterized by a high consumption of fruits, vegetables, nuts, cereals, legumes, fish, seafood, extra virgin olive oil and low intake of dairy products, meat and eggs [168]. The adherence to MD leads to decreased risk for metabolic, cardiovascular and chronic degenerative diseases, possibly due to the main components of MD which have anti-inflammatory properties [169,170,171,172]. Of all its components, extra virgin olive oil seems to play the most important role in its anti-inflammatory capacities [173]. A number of studies have highlighted the association between adherence to MD and psoriasis severity [174,175,176]. A case-control study by Barrea et al. was the first that showed that patients with psoriasis had a lower adherence to MD (assessed by PREMIDED questionary) compared to control group. In particular, a lower percentage of patients with psoriasis consumed extra virgin oil (as main lipid), fruits (≥3 portions/day), nuts (≥3 servings/week), fish or seafood (≥3 portions/week). Furthermore, psoriasis severity (assessed by PASI score and CRP) was negatively associated with the intake of extra virgin oil, fruits, nuts, fish or seafood, vegetables and legumes, whereas it was positively correlated with red meat intake [175]. In accordance with these results, a more recent cross-sectional observational study with a larger sample (*n* = 35.735 patients) by Phan et al. highlighted the inverse association between adherence to Mediterranean diet (assessed by MED-LITE) and psoriasis severity [176]. Interventional randomized clinical trials to confirm these results are still lacking. Based on the available studies, the Medical Board of the National Psoriasis Foundation recommends a trial of MD with the intake of extra virgin oil as a main lipid, 3 and 2 daily servings of fruits and vegetables respectively, 3 servings of fish/seafood, nuts, legumes weekly and 2 servings of sofrito sauce weekly in patients with psoriasis [167].

#### 3.2.3. Very-Low-Calorie Ketogenic Diet

In recent years, the ketogenic diet (KD) has been considered as an alternative diet strategy in managing obesity [177,178]. The classic ketogenic diet, very-low-carbohydrate ketogenic diet, Atkins diet, high-fat ketogenic diet and very low-calorie ketogenic diet (VLCKD) are different forms of KD. The main characteristic of KD is the low carbohydrate intake (less than 30–50 g/day) along with an increase in protein and fat [179]. This results in a metabolism switch to fat consumption as a main source of energy. Therefore, the ketogenic diet leads to an increase of fatty acids, ketone bodies and pyruvic acid. According to recent studies, the ketogenic diet has been considered as an alternative therapeutic strategy with beneficial results for many diseases such as obesity, type 2 diabetes, cardiovascular disease, neurological diseases and polycystic ovary syndrome [179,180,181,182,183]. The ketogenic diet improves oxidative stress via the activation of nuclear factor erythroid-derived 2 (NF-E2)—related factor 2 (Nrf2); its anti-inflammatory role is based on the activation of the peroxisome proliferator-activated receptor-gamma (PPAR-γ) and hydroxy-carboxylic acid receptor 2 (HCA2) [183,184,185]. Furthermore, β-hydroxybutyrate inhibits NLRP3 inflammasome in lipopolysaccharides (LPS)-stimulated human monocytes resulting in the reduction of IL-1β and IL-18 in a dose-dependent manner [186].

Based on this pathophysiological background, an increased interest regarding the effect of VLCKD on psoriasis exists, yet the evidence is still scarce. The first publication was a case report by Castaldo et al. about a 40-year-old female patient with recurrent moderate-to-severe plaque psoriasis, psoriatic arthritis and metabolic syndrome who was initially treated with biological therapy (adalimumab) for six months. At disease relapse the patient was treated with very-low-carb (300 Kcal/day), carbohydrate-free, high-protein (1.2 g per kilogram of ideal body weight) and low-fat diet as an add-on strategy to biological therapy. After 4 weeks, a significant reduction in PASI score >80% along with the resolution of psoriatic arthralgia were observed [187]. Recently, a single-arm, open-label clinical trial by Castaldo et al. evaluated the efficacy of a weight loss program with ketogenic diet as a first-line strategy in drug-naive patients with psoriasis. The intervention consisted of a 4-week VLCKD (<500 Kcal/d; 1.2 g of protein/Kg of ideal body weight/d) and a 6-week hypocaloric, low glycemic index, Mediterranean-like diet. This dietary intervention led to a body weight reduction of 12% and in a significant reduction of PASI score (mean change of −10.6) [188]. Similarly, another recent study investigated the effect of low-calorie ketogenic diet in 30 patients with psoriasis by evaluating clinical symptoms, biochemical markers, metabolomic profile and inflammatory markers (IL-2, IL-4, IL-1β, TNF-α, IFN-γ). Four-weeks of VLCKD resulted in 10% weight loss, 50% reduction in PASI score, improvement of biochemical markers related to psoriasis (folic acid, vitamin B12, cortisol, bilirubin, calcium, LDL, cholesterol) and decreased IL-1β and IL-2 levels [189]. Based on this data, ketogenic diet is a promising prospect in psoriasis management, but large studies are needed for safe conclusions.

### 3.3. Supplements and Psoriasis

#### 3.3.1. Vitamin D

As mentioned above, apart from being a calcium-regulating vitamin, vitamin D also exerts anti-inflammatory effects. The impact of 1,25(OH)2D3 on keratinocytes by inhibiting proliferation of hyperproliferative cells and increasing differentiation, together with its actions on T-cell activation, has rendered it a possible treatment option for patients with psoriasis [190,191]. According to recent data, patients with psoriasis have decreased vitamin D levels, and this possibly explains why the incidence of psoriatic disease is higher in locations less exposed to ultraviolet light, as this limited exposure leads to decreased vitamin D synthesis, together with the antiproliferative actions of ultraviolet radiation on keratinocytes [192,193]. Similarly, according to a case–control study, patients with psoriasis have lower 25-hydroxyvitamin D (25(OH)D) levels compared to the control group and are more likely to have 25(OH)D deficiency [194]. A possible explanation is that low vitamin D levels lead to the reduction of circulating T-cells and consequently to the dysregulation of immune balance [191]. For this reason, vitamin D and analogues have been proposed as possible treatments in psoriasis [190,195]. Topical therapy with vitamin D as an ointment or cream has been investigated thoroughly and the results indicate that vitamin D and analogues can be utilized either as monotherapy or in combination with a topical corticosteroid, methotrexate or cyclosporine [196,197,198]. The application of calcitriol ointment for eight weeks resulted in improvement of psoriatic lesions in approximately 34% of patients, compared with 12% to 22.5% of controls [197]. Even more interestingly, vitamin D and analogues have the same efficacy as topical corticosteroids without the adverse effect of skin atrophy, and they are thus suitable for long-term treatment. However, the results regarding the efficacy of oral vitamin D supplementation are inconsistent. A prospective study by Merola et al., including 70,743 female nurses who completed semi-quantitative food frequency questionnaires in 1994, 1998, 2004 and 2006, showed no association between vitamin D intake and the development of psoriasis [199]. Siddiqui et al. conducted a randomized, controlled, double-blinded trial including 50 patients who received 1 μg/day Vitamin D3 supplementation or placebo. No difference between the interventional and control group in psoriasis severity was revealed, implying that Vitamin D3 supplementation is not effective as a treatment for moderate to severe psoriasis [200]. Similarly, a more recent study by Ingram et al. included 101 patients who either received 100.000 IU Vitamin D3/month or placebo and evaluated the relationship between Vitamin D3 intake and psoriasis severity (assessed by PASI score). After three months, there was no significant difference in PASI score between the two groups [201]. In accordance with these results, another RCT with 23 patients who received supplementation with 100.000 IU Vitamin D/month and 42 controls showed that there was no significant difference in any of psoriasis outcome measures (PASI, PGA, PDI) after 12 months [202]. Gaal et al. showed that 0.5 μg/day Vitamin D supplementation had significant immunomodulatory effect in patients with polyarticular psoriasis [203]. Various hypotheses have been made to explain these discrepancies; a main reason is that vitamin D levels are not primarily determined by intake, but rather through sun exposure, a variable which is difficult to ensure in any patient group [201]. In addition, in some studies where no correlation between vitamin D and PASI was found, analysis was limited to only one measurement per person and not on repeated measurements over a follow-up period, while the relationship between PASI and 25(OH)D has not been shown to be linear, either. Lastly, not all patients equally respond to vitamin D administration, a fact which may be attributed to vitamin D receptor (VDR) polymorphisms. In conclusion, according to The Medical Board of the National Psoriasis Foundation, vitamin D supplementation is not recommended in patients with psoriasis and normal serum Vitamin D levels. However, patients with Vitamin D deficiency should receive Vitamin D oral supplements for prevention of psoriasis-related comorbidities [167].

#### 3.3.2. Vitamin B12

Vitamin B12 may have an impact on psoriasis through its contribution to nucleic acid synthesis. In vitro studies supported that Vitamin B12 regulates T-lymphocytes activation and cytokine secretion [204,205]. A few studies have reported an association between psoriasis and Vitamin B12 deficiency [206,207,208]. A retrospective observational study including 98 patients with psoriasis and 98 healthy individuals demonstrated that patients with psoriasis had increased homocysteine levels and lower serum levels of vitamin B12 and folic acid compared to healthy individuals [207]. In another report, the efficacy of vitamin B12 cream with avocado oil was investigated compared to the use of calcipotriol. After 8 weeks, treatment with calcipotriol had a more beneficial impact on psoriasis severity. However, after 12 weeks, no significant differences between the two treatment regimens were demonstrated [208]. Other studies have evaluated the impact of intramuscular administration of Vitamin B12 on the treatment of psoriasis with inconsistent results. Ruedemann et al. reported that the intramuscular administration of vitamin B12 for 10 days led to clinical improvement of psoriasis. In particular, in 11 out of 34 patients, the psoriatic lesions disappeared and 10 out of 34 reached 75% improvement in PASI score [209]. On the contrary, a double-blinded controlled study by Baker et al. showed that intramuscular administration of vitamin B12 for 3 weeks offered no benefit to psoriatic patients [210]. In general, vitamin B12 is not suggested as a treatment option for psoriasis.

#### 3.3.3. Polyunsaturated Fatty Acids

Research data regarding the effect of n-3 polyunsaturated fatty acids on the treatment of psoriasis are inconsistent. As oils of cold-water fish are rich in EPA and DHA, the effect of fish oil consumption and supplementation has been under investigation [211,212,213,214,215,216]. Collier et al. reported that daily intake of oily fish such as sardines, salmons, herrings and mackerels may promote clinical improvement. More specifically, the study included 18 patients who were advised to initially consume 170 g of white fish daily for 4 weeks and then they were randomized to consume either 170 g of white fish or 170 g of oily fish daily for six weeks; at the end of this second period, the diets were reversed for a further 6 weeks. The consumption of oily fish led to modest significant clinical improvement compared to white fish diet. Moreover, plasma EPA levels increased in patients with oily fish intake [211]. In 80 patients with chronic, stable psoriasis, 34 of whom also had psoriatic arthritis, supplementation with high doses of EPA and DHA for 8 weeks led to decreases in PASI score and a subjective improvement in joint pain [213]. Kragballe et al. reported that the cumulative consumption of 0.9 g/d EPA/DHA in 17 patients with psoriasis for 4 months led to moderate-excellent improvement in 10 of them; in a similar study, the supplementation at the dose of 1.9 g/d showed a significant decrease in PASI score after 4 and 8 weeks. On the contrary, an open study including 26 patients with psoriasis showed that fish oil supplementation did not improve psoriasis outcomes in any of the patients with plaque-type psoriasis except for one, a fact which was attributed to low dosage of EPA and the absence of dietary fat restriction [216].

The outcomes of randomized controlled trials are less encouraging [217,218,219,220]. In an RCT including 28 patients with stable chronic psoriasis, Bittiner et al. observed marked improvement of itching, erythema and scaling after 8 weeks of treatment with 3 g of n-3 fatty acids supplementation compared to olive oil supplementation [217]. On the other hand, two RCTs showed that 1.8 g of EPA for 8 weeks or 10 capsules of fish oil three times daily for three weeks did not achieve clinical improvement compared to olive oil supplementation [218,219]. Again, the unfavorable results can be possible explained by the low doses of EPA (1.8 g and 5.4 g daily) administered, together with the unrestricted dietary fat content, which leads to a lower concentration of EPA in cell membranes due to the competitive action of n-6 PUFAs. Søyland et al. conducted a 4-month double-blinded RCT, including 145 patients with moderate-to-severe psoriasis who were randomly allocated to either 6 g of fish oil daily or to isoenergetic corn oil containing mainly n-6 fatty acids. PASI score did not improve in either group, whereas scaling was reduced in both groups [220]. According to a recent review form Upala et al., including 12 studies, the results regarding the efficacy of n-3 PUFAs supplementation in the severity of psoriasis are still uncertain [221]. 

Clark et al. recently conducted a meta-analysis to investigate the efficacy of n-3 fatty acids as monotherapy in patients with psoriasis. The results showed that n-3 fatty acids led to significant reductions in PASI score, erythema, and scaling. In a subgroup analysis, higher dosages of >1800 mg/day and <8 weeks in duration were associated with more beneficial outcomes [222]. The results regarding the parenteral application of n-3 FAs are more encouraging compared to the mostly negative results of oral supplementation, probably because of the swift and sustainable increase in plasma levels of n-3 PUFASs leading to enhanced immune responses [223,224,225]. In an RCT by Grimminger et al., 20 patients were hospitalized for acute guttate psoriasis (BSA > 10%) and randomly received either an n-3 lipid emulsion (2.1 g EPA, 2.1 g DHA) or a conventional n-6 lipid emulsion for 10 days. In the n-3 treatment group, a greater improvement in all clinical manifestations (erythema, infiltration, desquamation) was noted [224]. These results were confirmed by another RCT with 83 patients hospitalized for chronic plaque-type psoriasis. After a 14-day treatment with the same emulsions, the PASI score decrease was greater in the n-3 group, an effect which was attributed to changes in eicosanoid regulation [225]. 

A few studies have investigated the efficacy of n-3 fatty acids supplementation in combination with other treatments for psoriasis [226,227,228]. An RCT including 18 patients with severe stable plaque psoriasis showed that fish oil supplementation in combination with UVB has beneficial effects on the clinical manifestations of psoriasis compared to the combination of olive oil with UVB [226]. Balbas et al. investigated the impact of a nutritional supplement rich in n-3 fatty acids (560 mg EPA, 80 mg DHA, Ovarex) in thirty patients with psoriasis, who received either 2 capsules of Ovarex daily as add-on treatment to topical tacalcitol or only topical treatment with tacalcitol (control group). After 8 weeks, the treatment group showed a significantly greater improvement in PASI score, in Nail Psoriasis Severity Index and in Dermatological Life Quality Index compared to controls [227]. Another study including 40 patients with psoriasis showed that oral etretinate treatment in combination with fish oil had better clinical outcomes on psoriatic manifestations compared to etretinate monotherapy. A >75% reduction in the clinical score was noticed in 45% in the intervention group compared to 15% in controls, and the mean duration to 50% reduction was 5.1 and 7.6 weeks, respectively [228]. Despite these promising results in general, large-scale trials are still needed to establish benefits for everyday clinical practice and, therefore, the Medical Board of the National Psoriasis Foundation does not include either oral or intravenous fish oil supplementation in the suggested dietary regimens [163].

#### 3.3.4. Selenium

Apart from the effects on oxidative stress, selenoproteins protect skin from harmful environmental factors such as ultraviolet (UV) rays, preventing keratinocytes apoptosis and increasing the cells’ ability to breakdown peroxides [229]. Decreased selenium levels along with the concomitant depressed selenium-dependent enzymatic activity have been observed in patients with psoriasis. Moreover, Serwin et al. reported that low selenium concentration is associated with increased severity of psoriasis in patients with disease duration more than three years [230]. Several studies have evaluated the efficacy of selenium supplementation in patients with psoriasis. Kharaeva et al. evaluated the efficacy of antioxidant supplementation in 58 hospitalized patients with severe erythrodermic and arthropathic forms of psoriasis. The authors reported that the combination of selenium aspartate, coenzyme Q10 and Vitamin E significantly improved psoriatic manifestations as assessed by PASI and Severity Score (symptom scoring for desquamation of plaques, plaque hyperemia, plaque inflammation, nail dystrophy, and joint pain) compared to placebo. Moreover, the activity of enzymes such as catalase and superoxide dismutase was diminished [231]. Juhlin et al. reported that selenium and Vitamin E supplementation for 8 weeks resulted in the increase of glutathione peroxidase in patients with psoriasis [232]. However, according to an older study in 69 patients, selenium and Vitamin E supplementation for 12 weeks did not reduce psoriasis severity [233]. Similarly, another case-control study, which included 37 patients, showed that selenium supplementation as add-on treatment to narrowband UVB therapy did not significantly improve psoriasis severity (assessed by PASI score) and TNF-R1 and CRP concentrations compared to placebo [234]. The same group examined the effect of selenium supplementation on TNF-R1 levels as add-on strategy to topical treatment with salicylic acid and dithranol ointment. The authors reported that despite the complete remission of skin lesions in both groups, the PASI score was higher in the treatment group, where the increased levels of TNF-R1 were also maintained [235]. These results implied that only inorganic Se compounds with cytostatic and cytotoxic activity may be of benefit to psoriasis patients; in addition, it was suggested that TNF-R1 reflects residual inflammation after clinical improvement of psoriatic lesions and that such high residual levels may be also attributed to the immunomodulating properties of selenium supplementation. As a result, a recommendation for selenium supplementation in patients with psoriasis cannot be established.

#### 3.3.5. Zinc

Only two RCTs have been conducted to investigate the efficacy of oral zinc supplementation in patients with psoriasis. In the study by Clemmensen et al. [236], including 34 patients with psoriatic arthritis, a 24-week zinc supplementation improved joint pain and mobility and alleviated joint swelling. Conversely, Burrows et al. showed that 12 weeks of treatment with zinc sulphate supplementation did not lead to significant differences in psoriasis and severity index in 25 patients with chronic plaque psoriasis [237].

#### 3.3.6. Polyphenols

The best studied phenolic compound regarding psoriasis is curcumin. [238]. Skyvalidas et al. suggested curcumin as a dietary immunosuppressant in patients with psoriasis due to in vitro inhibition of pro-inflammatory IFN-γ and IL-17 [239]. Furthermore, curcumin may enhance the secretion of anti-inflammatory cytokines such as IL-10 [240]. In a study by Antiga et al., curcumin supplementation was evaluated in 63 patients with moderate-to-severe psoriasis treated with topical steroids, who were randomly allocated to 2 g curcumin daily or placebo. After 12 weeks, the intervention group showed a greater improvement in median PASI score, along with a significant reduction in IL-22 serum concentration compared to the placebo group [241]. Bahraini et al. conducted an RCT including 40 patients with moderate-to-severe psoriasis, who were randomized to receive either turmeric tonic twice daily for nine weeks or placebo. Results demonstrated that turmeric tonic significantly improved erythema, scaling and induration of lesions compared to controls [242]. Another trial indicated that oral curcumin administration in combination with visible light phototherapy can improve moderate to severe plaque psoriasis [243], and a similar result was demonstrated with curcumin as an add-on therapy to acitretin [244]. On the contrary, a study from Kurd et al. showed that 4.5 g/d of oral curcuminoid C3 complex as monotherapy had no effect on any of the disease parameters [245]. In general, curcumin has shown promising results which, however, need to be confirmed by large-scale studies.

Data about other polyphenols is scarce and is mostly based on experimental animal models. In vitro studies have shown that resveratrol, through activating the SIRT1 pathway, can induce apoptosis in the HaCaT keratinocyte cell line and inhibit the production of IL-17 by Th-1 cells [246]. These actions have a favorable effect on psoriatic lesions. In a mouse model of imiquimod-induced psoriasis, resveratrol administration significantly diminished the severity of skin lesions and was associated with beneficial modifications in expression of retinoic acid stimulated genes, and IL-17A and IL-19 mRNA levels [247]. Another polyphenol with remarkable effects on psoriasis is epigallocatechin-3-gallate (EGCG), which is the most abundant catechin in green tea and is known to possess anti-inflammatory, antioxidant and antiproliferative properties [248]. In a study by Zhang et al. in BALB/c mice, which were topically treated with imiquimod for 6 consecutive days, treatment with EGCG attenuated skin inflammation and reduced skin infiltration of T cells, IL-17, IL-22, IL-23 and MDA levels, and increased SOD and CAT bioactivities [249]. Similarly, in mice with flaky skin, treatment with water and green tea extracts led to delayed and milder onset of skin lesions compared to the animals treated with water only [250], and in another mouse model of psoriasis, treatment with a nanoparticle formulation of EGCG led to 20-fold stronger therapeutic effect compared to free EGCG in terms of erythema, scales, infiltratory immune cells and pro-inflammatory cytokines [251]. However, the lack of human studies prevents any recommendation for these polyphenols to be applied as adjuvant treatment for psoriasis.

## 4. Conclusions

Experimental data both in vivo and in vitro, human epidemiological studies and randomized controlled trials have established the role of dietary components in chronic inflammation and oxidative stress. The adoption of Western-type diets with a high ratio of saturated fatty acids and a concomitant decrease in n-3 polyunsaturated fatty acids consumption, along with diets with high carbohydrate intake, lead to immune aberration and increased production of pro-inflammatory cytokines which are involved in the pathogenesis of many non-communicable diseases. On the other hand, micronutrients such as polyphenols and carotenoids seem to possess potent antioxidant properties, however, the high pharmacological doses needed to exert their effects cast doubt on their significance for everyday clinical practice. Instead, a diet rich in vegetables and fruits such as the Mediterranean diet seems to be the safest and most established pattern to prevent metabolic and immune derangement. Psoriasis is an immune-mediated disease where chronic inflammation plays a key role, a fact which has been consolidated through its established association with obesity, which negatively affects disease incidence, severity and response to treatment. Currently, low-calorie diets are the only dietary pattern with a proven benefit in all aspects of the disease and are practically considered to be a kind of adjuvant therapy to the conventional pharmacologic treatment. Gluten-free diets are highly recommended in patients with seropositivity for celiac disease; on the other hand, high-quality evidence for other strategies, such as ketogenic and Mediterranean diets or supplements are lacking, but they seem to have a promising potential. Indisputably, however, nutrition can be an additional therapeutic tool in psoriasis management, and this underlines the need for more, large-scale, randomized trials to confirm the beneficial effects of more dietary patterns and the underlying pathophysiological mechanisms, so as to understand which regimen fits each patient, and offer physicians and patients safe, feasible and individualized alternative approaches to alleviate the disease burden.

## Abbreviations and Acronyms

ABCA1	ATP-binding cassette transporter 1
ALA	a-linolenic acid
ARA	arachidonic acid
BMI	body mass index
BSA	body surface area
cAMP	cyclic adenosine monophosphate
CAT	catalase
CD	celiac disease
CI	confidence intervals
COX-2	cyclooxygenase-2
CRP	C-reactive protein
DHA	docosahexaenoic acid
eNOS	endothelial nitric oxide synthase
EPA	eicosapentaenoic acid
EVOO	extra virgin olive oil
FAs	fatty acids
FDS	freeze-dried strawberries
GL	glycemic load
GPR	G-protein coupled receptor
GPx	glutathione peroxidase
GRx	glutaredoxine
HCA2	hydroxy-carboxylic acid receptor 2
HDL	high-density lipoprotein
HFD	high fat diet
HR	hazard risk
ICAM-1	intracellular adhesion molecule 1
IFN-γ	interferon gamma
IL	interleukin
JAK-STAT	Janus Kinases—Signal Transducer and Activator of Transcription
KD	ketogenic diet
LA	linoleic acid
LDL	low-density lipoprotein
LPS	lipopolysaccharide
MCP-1	monocyte chemoattractant protein 1
MD	Mediterranean diet
MDA	malondialdehyde
MUFAs	monounsaturated fatty acids
NAFLD	nonalcoholic fatty liver disease
NCD	non-communicable diseases
NF-κB	nuclear factor-κB
NK	natural killer
NLRP3	NOD-like receptor protein 3
NO	nitric oxide
Nrf2	Nuclear factor erythroid 2-related factor 2
OA	oleic acid
PA	palmitic acid
PAI-1	plasminogen activator inhibitor-1
PASI	Psoriasis Area Severity Index
PGA	Physician’s Global Assessment
PGE2	prostaglandin E2
PPAR-γ	peroxisome proliferator-activated receptor-gamma
PUFAs	polyunsaturated fatty acids
RAR/RXR	retinoic acid receptor/retinoid X receptor
RCT	randomized controlled trial
ROS	reactive oxygen species
RR	relative risk
SAA	serum amyloid A
SCFAs	short-chain fatty acids
SFAs	saturated fatty acids
SIRT-1	sirtuin-1
SOD	superoxide dismutase
T2DM	type 2 diabetes mellitus
TCV	total caloric value
TG	triglycerides
Th	T-helper
TLR	Toll-like receptor
TNF-α	tumor necrosis factor alpha
tTG	tissue transglutaminase
UVB	ultraviolet B
VCAM-1	vascular cell adhesion molecule 1
VDR	vitamin D receptor
VLKCD	very low-calorie ketogenic diet
WAT	white adipose tissue
